# Continuously improve the medical care quality and hospital management level through medical information system construction

**DOI:** 10.1186/1479-5876-10-S2-A56

**Published:** 2012-10-17

**Authors:** Yunqing Qiu, Shuseng Zhen, Ming Zhou, Lanjuan Li

**Affiliations:** 1 The First Affiliated Hospital, School of Medicine, Zhejiang University, Hangzhou, 310003, China

## Background

The construction of medical information is important to improve the hospital medical care capability, the management decision-making level of health and the hospital operational efficiency. The construction of medical information system of the first Affiliated Hospital of Zhejiang University was begun in 1980s. Nowadays the comprehensive hospital information services and management platform have been established, centering on electronic medical records and clinical pathway. The establishment and use of these information systems played an important role in improving the degree of patient satisfaction, enhancing hospital efficiency and healthcare quality, protecting the safety of healthcare, and reducing healthcare costs.

## Materials and methods

Through the construction of clinical data repository (CDR) centering on electronic medical records, the integrated medical information system has been established in our hospital. At present, CDR has integrated the clinical diagnosis and treatment system,such as the electronic medical records system of inpatient and outpatient, nurse information system(NIS), laboratory information system (LIS), radiology information system (RIS), picture archiving and communication(PACS), ultrasonic information system (UIS), pathology information system(PIS), anesthesia management system, the remote medical network service platform, and the hospital outpatient and emergency registration system, outpatient appointment service system for disease diagnosis and treatment, physical equipment management system, cost management system, financial analysis system and the construction of hospital portal website.

## Results

The outpatient appointment rate of experts was more than 79%. The utilization ratio of hospital electronic medical records was almost 100%. The good quality medical record rate was over 98%. And overall efficiency of writing medical record was improved by 52.54%. The defect-free medical record rate was over 90%. The average hospital day was reduced by 7.8%. The 94 types of the clinical pathways were developed and applied in 24 departments. The remote medical network service platform was established with 90 hospitals.

## Conclusions

The establishment and application of hospital information systems improve hospital services and management standards, and ultimately improve the quality of medical services, reduce the medical errors, ensure the medical safety, and reduce healthcare costs.

